# Comparison of Echocardiography and Myocardial Scintigraphy to Detect Cancer Therapy-Related Cardiovascular Toxicity in Breast Cancer Patients

**DOI:** 10.3390/jimaging10030054

**Published:** 2024-02-21

**Authors:** Yuko Harada, Kyosuke Shimada, Satoshi John Harada, Tomomi Sato, Yukino Kubota, Miyoko Yamashita

**Affiliations:** 1 Department of Cardiology, Kawasaki Municipal Ida Hospital, Kawasaki 211-0035, Japan; 2 Department of Breast Surgery, Kawasaki Municipal Ida Hospital, Kawasaki 211-0035, Japan; 3 School of Medicine, Shinanomachi Campus, Keio University, Tokyo 160-8582, Japan; 4 Department of Palliative Care Medicine, Kawasaki Municipal Ida Hospital, Kawasaki 211-0035, Japan; 5 Department of Radiology, Kawasaki Municipal Ida Hospital, Kawasaki 211-0035, Japan

**Keywords:** breast cancer, cancer-therapy-related cardiovascular toxicity (CVR-CVT), chemotherapy-related cardiac dysfunction (CTRCD), ^123^I-BMIPP, Tl/BMIPP dual-isotope myocardial scintigraphy

## Abstract

The mortality rate of cancer patients has been decreasing; however, patients often suffer from cardiac disorders due to chemotherapy or other cancer therapies (e.g., cancer-therapy-related cardiovascular toxicity (CVR-CVT)). Therefore, the field of cardio-oncology has drawn more attention in recent years. The first European Society of Cardiology (ESC) guidelines on cardio-oncology was established last year. Echocardiography is the gold standard for the diagnosis of CVR-CVT, but many breast cancer patients are unable to undergo echocardiography due to their surgery wounds or anatomical reasons. We performed a study to evaluate the usefulness of myocardial scintigraphy using Iodine-123 β-methyl-P-iodophenyl-pentadecanoic acid (^123^I-BMIPP) in comparison with echocardiography and published the results in the Journal of Imaging last year. This is the secondary analysis following our previous study. A total of 114 breast cancer patients who received chemotherapy within 3 years underwent echocardiography, as well as Thallium (^201^Tl) and ^123^I-BMIPP myocardial perfusion and metabolism scintigraphy. The ratio of isotope uptake reduction was scored by Heart Risk View-S software (Nihon Medi-Physics). The scores were then compared with the echocardiography parameters. All the patients’ charts and data from January 2022 to November 2023 were reviewed for the secondary analysis. Echocardiogram parameters were obtained from 99 patients (87% of total patients). No correlations were found between the echocardiography parameters and Heart Risk View-S scores of ^201^Tl myocardial perfusion scintigraphy, nor those of the BMIPP myocardial metabolism scintigraphy. In total, 8 patients out of 114 (7.0%) died within 22 months, while 3 patients out of 26 CVR-CVT patients (11.5%) died within 22 months. Evaluation by echocardiography was sometimes difficult to perform on breast cancer patients. However, other imaging modalities, including myocardial scintigraphy, cannot serve as alternatives to echocardiography. Cardiac scintigraphy detects circulation disorder or metabolism disorder in the myocardium; therefore, it should be able to reveal myocardial damage to some extent. The mortality rate of breast cancer patients was higher with CVR-CVT. A new modality to detect CVR-CVT besides echocardiography can possibly be anticipated for patients who cannot undergo echocardiography.

## 1. Introduction

Cancer therapies have advanced greatly in recent years, and thus, cancer patients survive longer than before. However, they often develop heart disease or vascular disease after chemotherapy or radiotherapy. Such cardiovascular disease is called cancer-therapy-related cardiovascular toxicity (CVR-CVT) [1]. This research field is called cardio-oncology or onco-cardiology and is rapidly growing worldwide. Associations for cardio-oncology have been established in the Western and Eastern worlds.

The first European Society of Cardiology (ESC) guidelines on cardio-oncology were established in November 2022 [1]. The predecessor was the ESC Position Paper published in 2016 [2]. The American Heart Association (AHA) statement published in 2019 was also regarded as an alternative to the guidelines [3]. Several terminologies and definitions were proposed to describe CVR-CVT and the term cardio-oncology was integrated into CVR-CVT in the ESC guidelines. In the Position Paper, the term cancer-therapeutic-related cardiac dysfunction (CTRCD) was used, but the term CTRCD was recommended to be used only for cardiac injury, cardiomyopathy, and heart failure in the ESC guidelines. According to the new ESC guidelines, CVR-CVT includes CTRCD (symptomatic and asymptomatic), immune checkpoint inhibitors myocarditis, vascular toxicity, arterial hypertension, and cardiac arrhythmias [1]. A variety of cardiovascular diseases due to cancer therapy is regarded as CVR-CVT.

Echocardiography is recommended as the first-line modality for the assessment of cardiac function in patients with cancer in the ESC guidelines and Position Paper [1,2]. Echocardiography is non-invasive and useful for most patients but is difficult to perform on those who have surgery wounds and scars in the chest. It is also difficult to perform echocardiography on patients with obesity, funnel chest, and pigeon chest. In the ESC guidelines, the second-line recommendation for the imaging modality is cardiac MRI (CMR), and the third-line recommendation is multigated radionuclide angiography (MUGA). However, not all hospitals have CMR, as it requires special settings that are different from classical MRI. MUGA is available if the facility has a camera for scintigraphy. The advantage of MUGA is its reproducibility, while its limitations are cumulative radiation exposure, as well as its limited structural and functional information beyond the left ventricle ejection fraction (LVEF) [4]. MUGA can be performed in place of echocardiography to measure the LVEF, but imaging with an isotope that can evaluate the damage of the myocardium at the cell level is anticipated. We, therefore, selected a new modality, namely, myocardial scintigraphy using Iodine-123 β-methyl-P-iodophenyl-pentadecanoic acid (^123^I-BMIPP), to evaluate CVR-CVT for patients who could not undergo echocardiography. Myocardial scintigraphy with ^123^I-BMIPP has been widely used in Japan to detect myocardial damage, especially for ischemic heart disease and cardiomyopathy [5,6,7]. CVR-CVT due to chemotherapy is medically the same as drug-induced cardiomyopathy; therefore, CVR-CVT can be detected by myocardial scintigraphy. ^123^I-BMIPP is a tracer for the metabolism of fatty acid, which is known to account for more than 90% of myocardial energy requirements [8]. ^123^I-BMIPP is typically utilized in dual-isotope myocardial scintigraphy with 201-Thallium (^201^Tl), which is called Tl/BMIPP dual-isotope myocardial scintigraphy and is used to evaluate any accumulation mismatch [5,6]. The process of scintigraphy involves using both ^201^Tl and ^123^I-BMIPP, while solo scintigraphy of ^123^I-BMIPP does not exist. The limitation of this modality is higher radiation exposure than MUGA.

In November 2022, we published our study that evaluated the usefulness of myocardial scintigraphy using ^123^I-BMIPP in comparison with echocardiography [9]. We focused on breast cancer patients because many of them are unable to undergo echocardiography due to surgery wounds and implants in their chest. It is also known that shortfalls in echocardiography are caused by constraints due to the dependency on acoustic windows and variable operator skills [10].

In the previous study, we compared LVEF and Thallium (^201^Tl) uptake scores; however, correlations were not observed. We also compared LVEF and ^123^I-BMIPP, but there was also no correlation. Then, a new question was raised: “Is myocardial scintigraphy able to detect diastolic dysfunction caused by CVR-CVT?” After publication, our team performed an additional study for secondary analysis using the data collected in the previous study. We also performed a post-publication retrospective study to evaluate mortality in the participants.

In this study, we compared the echocardiography parameters of diastolic dysfunction and ^201^Tl/^123^I-BMIPP uptake in dual myocardial scintigraphy. To evaluate the diastolic function of the heart, several parameters were used in combination: E wave, E/A ratio, septal or lateral e’, average E/e’, left atrial volume index, and peak tricuspid regurgitation velocity [11,12,13]. In this study, E/A, septal e’ (e’ in this article), and average E/e’ were chosen for convenience. The other parameters, such as left atrial volume index and peak tricuspid regurgitation velocity, were not calculated as routine check-ups due to the limitation of testing time. Another parameter of systolic function, namely, left ventricular systolic diameter (LVSD), was also evaluated in addition to the previous study for systolic function using LVEF.

The originality of this study, as well as of our previous paper, was that we uniquely utilized the computerized scores (Heart Risk View-S scores) to quantify the images for myocardial scintigraphy, thereby enabling quantitative comparison with echocardiography. The advantage of echocardiography in the diagnosis of CTRCD is that echocardiography is quantified with a variety of parameters to enable easy comparison with other patients. Each echocardiogram parameter has a universal normal range, thereby enabling the precise diagnosis of CTRCD. On the other hand, myocardial scintigraphy has been devoid of any such quantitative parameters, thereby requiring non-quantitative (blurry) visual eyeballing by nuclear cardiologists. Experienced nuclear cardiologists visually attempt to estimate the percentage of isotope uptake reduction, thereby virtually creating a score from 0 to 4 in each section of the planes. Special note was taken of such a scoring system for myocardial scintigraphy, which would precisely quantify the images with computer software. This is similar to the parameterization of echocardiography. Utilizing such a scoring system thereby enabled us to uniquely quantitatively compare echocardiography and myocardial scintigraphy images. This method is unique and has never been reported thus far to the best of our knowledge.

## 2. Materials and Methods

A total of 114 histologically confirmed breast cancer patients who received chemotherapy for at least 2 months within 3 years were eligible for this study. Those who were not able to communicate were not included, as were those unable to lay on their backs nor remain still. Terminal-stage cancer patients were also excluded, as they were not eligible for the medical benefits for this costly testing. All were outpatients of the Department of Breast Surgery at Kawasaki Municipal Ida Hospital, and they provided written consent to be enrolled in this study. Regulatory approval for this study was granted by the research ethics board of Kawasaki Municipal Ida Hospital.

Anti-cancer drugs were given in accordance with the guidelines of the Japanese Breast Cancer Society [14]. All patients underwent echocardiography prior to chemotherapy and every 6 months during chemotherapy. The most recent data of echocardiography recorded in patients’ charts were collected. The LVEF and GLS were evaluated in the previous study. The parameters collected in this study were E/A, E/e’, e’, and left ventricle systolic diameter (LVSD).

Tl/BMIPP dual-isotope myocardial scintigraphy was performed on an outpatient basis. Patients fasted for at least 6 h prior to the study, then were intravenously injected with 111 Mbq of ^123^I-BMIPP (Nihon Medi-Physics Co., Ltd., Tokyo, Japan). Single-photon emission computed tomography (SPECT) images were acquired starting 15 min after the tracer injection using a digital gamma camera Symbia E (Canon Medical Systems Corporation, Tochigi, Japan).

Myocardial SPECT images were then analyzed by the automated software program Heart Risk View-S (Nihon Medi-Physics Co., Ltd., Tokyo, Japan). The software generated polar maps of the heart from myocardial SPECT data that were divided into 17 segments, as recommended by the guidelines of the American Heart Association (called the 17-segment model) [15]. The software first calculated the mean percentage uptake count for each segment and deployed the percentage in the 17-segment model. These percentages were then compared with the normal ^201^Tl and ^123^I-BMIPP database developed for Japanese patients by the Japanese Society of Nuclear Medicine working group [16,17,18]. The mean percentage uptake count for each segment was then recalculated for the express purpose of eliminating the effect of artifacts and converted to scores using a five-point scale from normal to absent (0, normal; 4, absent). These recalculated Heart Risk View-S scores were then deployed in the 17-segment model as the final result. Segments with 60–70%, 50–60%, and 40–50% uptakes were classified as mild, moderate, and severe uptake reductions, and thereby scored as 1, 2, and 3, respectively. Segments with uptake less than 40% were scored as 4. Segments with normal uptake were scored as 0.

Heart Risk View-S scores of ^201^Tl uptake and ^123^I-BMIPP uptake were compared with each echocardiogram parameter for each patient. Echocardiogram parameters were not fully available for 15 patients; therefore, 99 patients (87% of the total 114 patients) were evaluated for comparison with ^201^Tl uptake. Among these 99 patients, 2 patients revealed no uptake of ^123^I-BMIPP, which were excluded as outliers. The data of each echocardiogram parameter and the Heart Risk View-S scores of ^201^Tl and ^123^I-BMIPP were deployed in scatter plots, and the Pearson product-moment correlation coefficient was calculated.

All the data and charts of 114 participants were thoroughly reviewed to evaluate the morbidity of CTRCD and mortality.

## 3. Results

### 3.1. Tl/BMIPP Dual-Isotope Myocardial Scintigraphy

**Figure 1:**
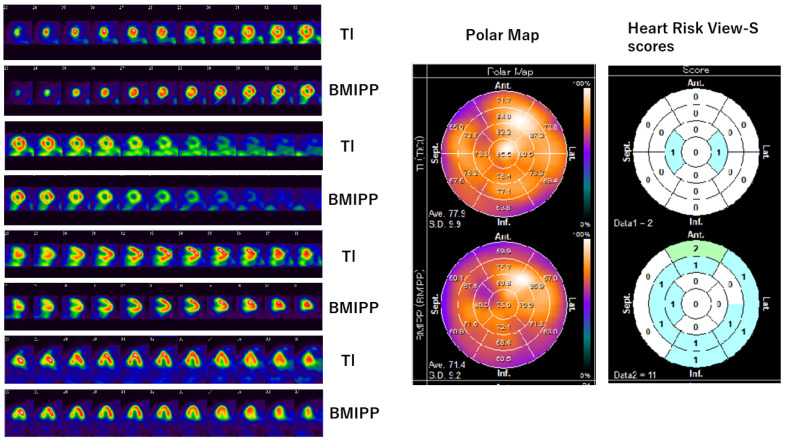
Tl/BMIPP dual-isotope myocardial scintigraphy of a patient. (**Left**) SPECT images are arranged in order from top to bottom: left ventricular short-axis planes of ^201^Tl and ^123^I-BMIPP, long-axis planes of ^201^Tl and ^123^I-BMIPP, and horizontal planes of ^201^Tl and ^123^I-BMIPP. (**Middle**) Polar map of ^201^Tl (**top**) and ^123^I-BMIPP (**bottom**). (**Right**) Heart Risk View-S scores of ^201^Tl (**top**) and ^123^I-BMIPP (**bottom**). Patient E in Table 2. Original image data can be found in Supplementary Materials (Figures S3 and S4).

**Figure 2:**
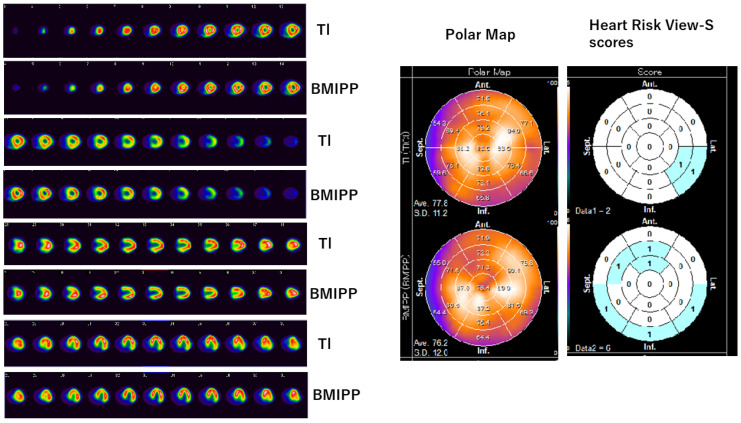
Tl/BMIPP dual-isotope myocardial scintigraphy of another patient. (**Left**) SPECT images are arranged in order from top to bottom: left ventricular short-axis planes of ^201^Tl and ^123^I-BMIPP, long-axis planes of ^201^Tl and ^123^I-BMIPP, and horizontal planes of ^201^Tl and ^123^I-BMIPP. (**Middle**) Polar map of ^201^Tl (**top**) and ^123^I-BMIPP (**bottom**). (**Right**) Heart Risk View-S scores of ^201^Tl (**top**) and ^123^I-BMIPP (**bottom**). Patient H in Table 2. Original image data can be found in Supplementary Materials (Figures S7 and S8).

Myocardial scintigraphy was performed for all the 114 patients enrolled in this study. The SPECT images, polar maps, and Heart Risk View-S scores obtained are shown in Figure 1 and Figure 2.

Figure 1 shows data from a 59-year-old patient who underwent surgery and chemotherapy. Implants were installed after surgery, which obstructed the ultrasound and echocardiography was difficult. She received multiple anti-cancer drugs, such as Epirubicin, Trastuzumab, Bevacizumab, and Paclitaxel. SPECT images and polar maps of Figure 1 reveal mild ^201^Tl uptake reduction in the anterior wall of the heart, as well as ^123^I-BMIPP uptake reduction in the anterior wall, lateral wall, and inferior wall. The Heart Risk View-S scores were 2 for ^201^Tl and 11 for ^123^I-BMIPP. The ^201^Tl uptake reduction in the anterior wall could have been caused by an artifact from the implant. However, the ^123^I-BMIPP uptake was not affected by the implants. Since the ^123^I-BMIPP uptake decreased more than the ^201^Tl uptake, the myocardial metabolism was impaired more than myocardial perfusion in this patient. As the ^123^I-BMIPP uptake was diffusely decreased, CVR-CVT (i.e., CTRCD) was suspected. The patient was asymptomatic at the enrollment of the study, but she died of multiple organ failure in 11 months.

Figure 2 shows data from a 75-year-old patient who underwent surgery and chemotherapy, but she did not receive implants. She also received multiple anti-cancer drugs, such as Epirubicin, Trastuzumab, and Paclitaxel. SPECT images and polar maps of Figure 2 reveal mild ^201^Tl uptake reduction in the lateral wall and apex, as well as ^123^I-BMIPP uptake reduction in the anterior wall, lateral wall, and apex. Heart Risk View-S scores were 2 for ^201^Tl and 6 for ^123^I-BMIPP. ^123I^I-BMIPP uptake was also diffusely decreased in this patient; therefore, CVR-CVT (i.e., CTRCD) was suspected. The patient was asymptomatic at this point; however, the disease developed rapidly and she died in 3 months.

### 3.2. Echocardiography

**Figure 3:**
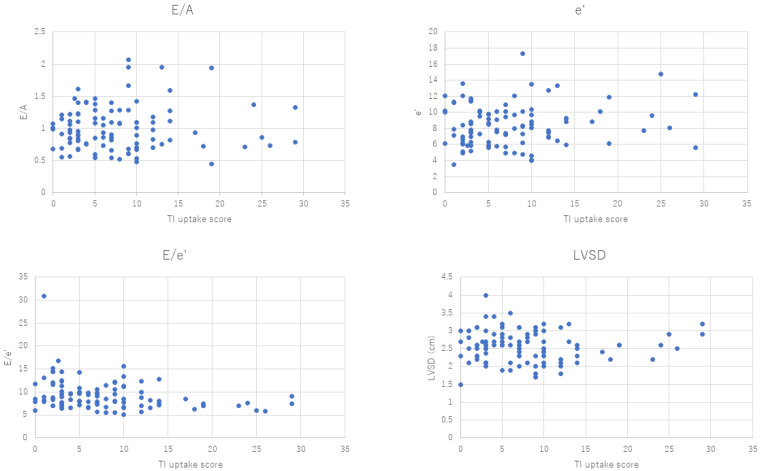
Scatter plots of ^201^Tl uptake (horizontal axis) and E/A, e’, E/e’, and LVSD.

Echocardiography parameters were fully obtained from 99 patients out of 114 (87% of total patients) and not fully available from 15 patients (13.2%) due to anatomical factors, surgical wounds, or implants. The ^201^Tl uptake and ^123^I-BMIPP uptake were compared with E/A, E/e’, e’, and LVSD. The Heart Risk View-S scores and each echocardiogram parameter of each patient are presented in scatter plots. Figure 3 is the comparison of ^201^Tl uptake and echocardiography parameters. The correlation was calculated using the scatter plots.

**Figure 4:**
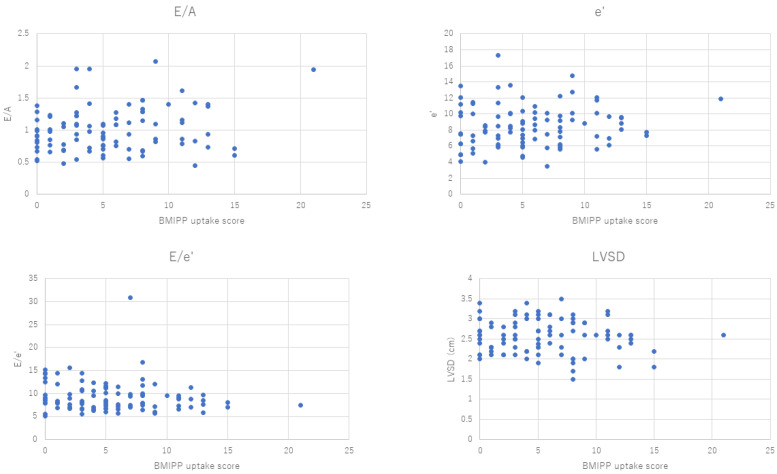
Scatter plots of ^123^I-BMIPP uptake (horizontal axis) and E/A, e’, E/e’, and LVSD.

Figure 4 is the comparison of ^123^I-BMIPP uptake and echocardiography parameters. Two cases revealed ^123^I-BMIPP uptake scores of 64 and 68, which were too high compared with the others. They were regarded as outliers.

The Pearson product-moment correlation coefficient (*p*-value) ranged from −0.28 to 0.2, as shown in Table 1. The *p*-values were close to 0, which implied that there was no correlation between the Heart Risk View-S scores and the parameters of the echocardiogram.

### 3.3. Morbidity and Mortality

All the data and charts of 114 participants were thoroughly reviewed. Six patients were diagnosed with CTRCD by previous ESC Position Paper criteria of decreased LVEF below 53% [2]. These patients are classified as “CTRCD patients by LVEF criteria” in Table 2.

Twenty patients showed decreased ^123^I-BMIPP uptake below 50% (Heart Risk View-S score 2 and higher in each segment), which suggested certain damage in the myocardium. These patients were classified as “Possible CTRCD patients by scintigraphy” in Table 2. Combined with these two groups, the morbidity of CTRCD in our study was 22.8% (26 patients out of 114).

A total of eight patients passed away in the 22 months since our previous study started (Table 2). The patients’ ages ranged from 40 to 75, and the mean age was 58. The duration from scintigraphy to death ranged from 3 months to 20 months, and the mean duration was 6 months.

The mortality rate was 8/114 (7.0%) in total and 3/26 (11.5%) in CTRCD and “Possible CTRCD” patients (Table 2). Note that only one patient diagnosed with CTRCD by LVEF criteria passed away. Most of the deceased patients were not diagnosed with CTRCD by the criteria but showed high ^123^I-BMIPP scores. All patients died within only 22 months, even though all the patients were asymptomatic at the enrollment of the study. The cause of death of eight patients was multiple organ failure due to advanced cancer in most cases and interstitial pneumonia in two patients. 

All the raw data of patient C,E,G and H are shown in Figures S1–S8 of Supplementary Materials. Data of patient X and patient Y who recovered from CTRCD are also shown in Figures S9–S12 of Supplementary Materials for reference.

## 4. Discussion

In our previous study, a comparison between LVEF and Heart Risk View-S scores also did not reveal any correlation. Our speculation for the reason was that the cardiac damage was mild or at an early stage in our patients because all the patients were asymptomatic. Our conclusions were that ^123^I-BMIPP myocardial metabolism scintigraphy revealed possible early signs of CTRCD for breast cancer patients [9]. It appeared that Tl/BMIPP dual-isotope myocardial scintigraphy was not strongly correlated with cardiac pump failure due to CTRCD.

In this current study, we compared each isotope uptake and echocardiogram parameters for diastolic dysfunction. However, the ^201^Tl uptake scores and ^123^I-BMIPP uptake scores did not reveal a close relationship with E/A, E/e’, e’, and LVSD. Therefore, Heart Risk View-S scores are probably not related to diastolic dysfunction of the heart.

An important confounding factor of myocardial scintigraphy is that ^201^Tl myocardial perfusion scintigraphy is subjected to artifacts from the breast and liver [19,20]. As shown in Figure 1, a thick chest wall or implants in the chest may cause artifacts in SPECT figures of ^201^Tl.

Two cases revealed ^123^I-BMIPP uptake scores of 64 and 68, which were too high compared with the others. As these scores were near the maximum value (full score was 68) of the tests, we regarded them as outliers. These patients were asymptomatic in spite of maximum uptake scores, which suggested that they had some disorders in ^123^I-BMIPP uptake, such as CD36 deficiency or triglyceride deposit cardiomyovasculopathy (TGCV) [20,21,22].

Consistent with our previous and current studies, the Heart Risk View-S scores were not related to echocardiography parameters. This implied that Tl/BMIPP dual-isotope myocardial scintigraphy did not reveal the systolic or diastolic function of the heart. Therefore, Tl/BMIPP dual-isotope myocardial scintigraphy cannot serve as an alternative to echocardiography. Echocardiography evaluates the kinetic factors of the heart, whereas myocardial scintigraphy evaluates the perfusion and metabolism of the heart.

When we are asked whether Tl/BMIPP dual-isotope myocardial scintigraphy is useful for detecting CVR-CVT, we do not have the answer yet. Tl/BMIPP dual-isotope myocardial scintigraphy does in fact reveal myocardial damage, and it has been used for decades. Therefore, myocardial scintigraphy should be useful for detecting myocardial damage due to cancer therapies, especially for patients who cannot undergo echocardiography. There are only a few reports regarding ^123^I-BMIPP scintigraphy performed for CTRCD, but the numbers of patients in these studies ranged from only 13 to 26 [8,23,24]. Tl/BMIPP dual-isotope myocardial scintigraphy has not yet been considered as a tool to evaluate CVR-CVT. A multi-center prospective study is necessary. It may take some time until the tracer ^123^I-BMIPP is widely recognized, as it is not yet widely used in practice. For example, it has not yet been approved by the Food and Drug Administration (FDA) in the USA [25]. Tl/BMIPP myocardial scintigraphy is recommended in class IIb for diagnosis of vasospastic angina in Japanese guidelines, but it is not yet referred to in Western guidelines [26,27]. However, ^123^I-BMIPP has been introduced as a promising new tracer in reviews internationally [4,10,25]. ^123^I-BMIPP scintigraphy (or Tl/BMIPP dual-isotope myocardial scintigraphy) may have other roles than echocardiography in evaluating CVR-CVT.

For cancer patients with cardiovascular risk factors, such as hypertension, diabetes, aging, and smoking, it is sometimes difficult to determine the cause of cardiovascular disease. It could be caused by cancer therapy, but it may also be caused by ischemic heart disease with atherosclerosis, arrhythmia, cardiac valvular disease, or idiopathic cardiomyopathy. Currently, it is difficult to show which factor (or which agent) affected the myocardium or vessels to cause cardiovascular diseases in cancer patients. In this regard, myocardial scintigraphy is more useful than echocardiography for identifying drug-induced cardiomyopathy, including CTRCD, because scintigraphy can visualize the degree and the area of myocardial damage. If the isotope uptake is decreased in the perfusion area of each coronary artery, the diagnosis is ischemic heart disease. If the isotope uptake is decreased diffusely regardless of coronary perfusion, the diagnosis will be non-ischemic cardiomyopathy. This method with myocardial scintigraphy will be useful to detect CVR-CVT.

The morbidity of CTRCD was 22.8% (5.3% by LVEF criteria and 17.5% by scintigraphy). The 22-month mortality was 7.0% in this study. All patients revealed no signs of heart disease at the enrollment of the study. Patients passed away at a rather younger age (mean age 58 years old) and within a rather short period (mean duration 6 months after scintigraphy). Two patients died of interstitial pneumonia due to anti-cancer drugs or radiation therapy, and the other patients died of multiple organ failure due to advanced cancer. However, no patient underwent an autopsy, and thus, the cause of death was unknown. It may have been affected by CVR-CVT (or CTRCD), but it is difficult to show this after death.

After the previous study, the breast surgeons changed the chemotherapy procedure for patients with “CTRCD by LVEF criteria” and “Possible CTRCD by scintigraphy”. This may have affected the mortality of these 26 patients. A change in chemotherapy may have accelerated the development of cancer. However, if the chemotherapy had not been changed, the anti-cancer drug would have damaged the heart to accelerate the development of heart failure. Clinicians want to know “what to do next” after a diagnosis of CTRCD. It is not known how to change the procedure for CTRCD patients. Most patients are treated with multiple drugs that are known to be cardiotoxic. These drugs are “double-edged swords” that are quite effective against cancer on the one hand and toxic to the heart on the other. Almost all the anti-cancer drugs for breast cancer are cardiotoxic. In the future, the anticipated guidelines for CVR-CVT should also include how to treat CVR-CVT or how to change the chemotherapy procedure.

The mortality of breast cancer in Japan is 12.0 (men 0.2, women 23.1) per 100,000 population [28]. The 5-year survival is 92.3% [28]. The 22-month mortality is not reported, but the 5-year mortality is calculated to be 7.6%, which is comparable with our 22-month mortality. According to reports from the World Health Organization, the death rate of breast cancer in the age group 55–74 is 39 per 100,000 population [29]. The Israel Cardio-oncology registry reported that 5% of breast cancer patients developed CTRCD according to ESC Position Paper criteria, which is similar to our results [30]. The mortality of breast cancer patients is high, and the morbidity of CTRCD is also high. Therefore, there is an urgent need to establish universal guidelines for CVR-CVT for breast cancer patients.

This study had some limitations. First, we could not compare the myocardial scintigraphy results before and after chemotherapy due to the different medical insurance policies. Therefore, we needed to compare data after chemotherapy with the database in the Heart Risk View-S software. It would, however, have been preferable if scintigraphy results were available prior to chemotherapy for each patient. Second, the durations and procedures of chemotherapy were different for each patient. Some patients had undergone chemotherapy for years, while others had it for only a few months. It is thus difficult to find patients with parallel conditions of chemotherapy at the same time. Third, the timings of the echocardiography and myocardial scintigraphy were not the same. The time differences varied from weeks to several months due to medical insurance policies. If the tests were able to be performed at the same time, the results might have been somewhat different.

Some nuclear cardiologists may not want to use software to score myocardial scintigraphy, as the software does not exclude artifacts. Therefore, all the SPECT images must be visually evaluated before scoring. Heart Risk View-S scores are calculated based on the database of 6000 Japanese patients, but they may not apply to non-Japanese patients. The scores may not be accurate for ^201^Tl because the software cannot identify artifacts from the chest wall or liver. The scores also may not be accurate for ^123^I-BMIPP when the patient has a CD36 deficiency or TGCV. However, the software is very helpful for non-specialists who want to detect myocardial diseases, including CTRCD (or CVR-CVT). The diagnostic criteria for CTRCD should be universal, reproductive, and quantitative. Such software to calculate scintigraphy scores is a desirable diagnostic tool for physicians or surgeons who are not familiar with nuclear cardiology. As ^123^I-BMIPP scintigraphy is not widely used in practice, it will take some time to collect a database for all races and nations to build universal software for calculating ^123^I-BMIPP uptake reduction scores. If a single scintigraphy measurement of ^123^I-BMIPP and its uptake reduction scoring system are established, they will become very useful for the evaluation of CVR-CVT.

Most research papers about ^123^I-BMIPP are written in the Japanese language. ^123^I-BMIPP has a long history in Japan, and nearly all the evidence is confined to Japan. Yet, even in Japan, not all hospitals use ^123^I-BMIPP. If ^123^I-BMIPP is commonly used, the isotope uptake reduction scoring system would be improved with a larger database and the scores would become even more reliable and precise.

## 5. Conclusions

Evaluation by echocardiography is sometimes difficult for breast cancer patients. However, there are no acceptable alternative imaging modalities. Quantitative comparisons with myocardial scintigraphy and echocardiography were performed utilizing Heart Risk View-S scores and echocardiogram parameters. However, there were no correlations between them. Therefore, myocardial scintigraphy may not serve as an alternative to echocardiography. Myocardial scintigraphy detects a circulation disorder or metabolism disorder in the myocardium; therefore, it should be able to reveal myocardial damage to some extent.

The mortality rate of breast cancer patients was higher with CVR-CVT (or CTRCD). A new modality to detect CVR-CVT in addition to echocardiography is anticipated. Myocardial scintigraphy, especially with ^123^I-BMIPP, is therefore a promising modality if it is used worldwide. The establishment of guidelines on how to treat CVR-CVT is anticipated.

## Figures and Tables

**Table 1:** *p*-values calculated from scatter plots in Figure 3 and Figure 4.

**Isotope**	**E/A**	**e’**	**E/e’**	**LVSD (cm)**
^201^Thallium	0.045024	0.128372	−0.27713	−0.01576
^123I^I-BMIPP	0.19986	0.121109	−0.1199	−0.08403

**Table 2:** Patients passed away during study (images can be found in Supplementary Materials).

**Patient**	**Age**	**^201^** **Tl Score**	**^123^** **I-BMIPP Score**	**Duration to Death (Months)**	**CVR-CVT**	**Cause of Death**
A	40	2	1	20	-	Multiple organ failure
B	52	10	5	6	-	Multiple organ failure
C	55	9	22	3	Possible CTRCD patient by scintigraphy	Interstitial pneumonia
D	58	3	8	5	-	Multiple organ failure
E	59	2	11	11	Possible CTRCD patient by scintigraphy	Multiple organ failure
F	68	12	7	10	-	Multiple organ failure
G	72	3	68	6	CTRCD patient by LVEF criteria	Multiple organ failure
H	75	2	6	3	-	Interstitial pneumonia

## Data Availability

Data supporting reported results can be found at Kawasaki Municipal Ida Hospital.

## Supplementary Materials

The following supporting information can be downloaded from https://www.mdpi.com/article/10.3390/jimaging10030054/s1, Figure S1: ^201^Tl SPECT of patient C in Table 2; Figure S2: ^123^I-BMIPP SPECT of patient C in Table 2; Figure S3: ^201^Tl SPECT of patient E in Table 2; Figure S4: ^123^I-BMIPP SPECT of patient E in Table 2; Figure S5: ^201^Tl SPECT of patient G in Table 2; Figure S6: ^123^I-BMIPP SPECT of patient G in Table 2; Figure S7: ^201^Tl SPECT of patient H in Table 2; Figure S8: ^123^I-BMIPP SPECT of patient H in Table 2; Figure S9: ^201^Tl SPECT of patient X who recovered from CTRCD; Figure S10: ^123^I-BMIPP SPECT of patient X who recovered from CTRCD; Figure S11: ^201^Tl SPECT of patient Y who recovered from CTRCD; Figure S12: ^123^I-BMIPP SPECT of patient Y who recovered from CTRCD.
