# Supplementary figures and images for: Comparison of Echocardiography and Myocardial Scintigraphy to Detect Cancer Therapy-Related Cardiovascular Toxicity in Breast Cancer Patients
Source: J Imaging. 2024 Feb 21;10(3):54. doi: 10.3390/jimaging10030054 (PMC10971125; doi:10.3390/jimaging10030054)

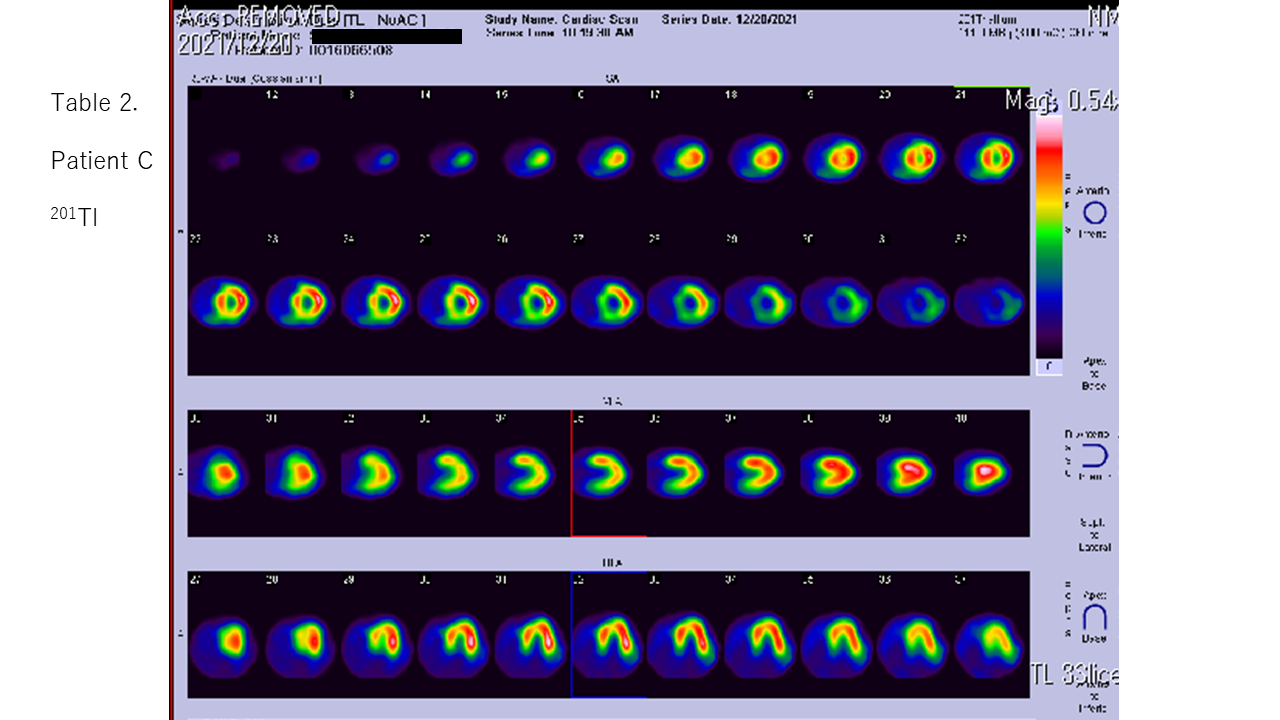

Supplement: Supplementary file 1 [file jimaging-10-00054-s001.zip › Supplement file 1.tif]

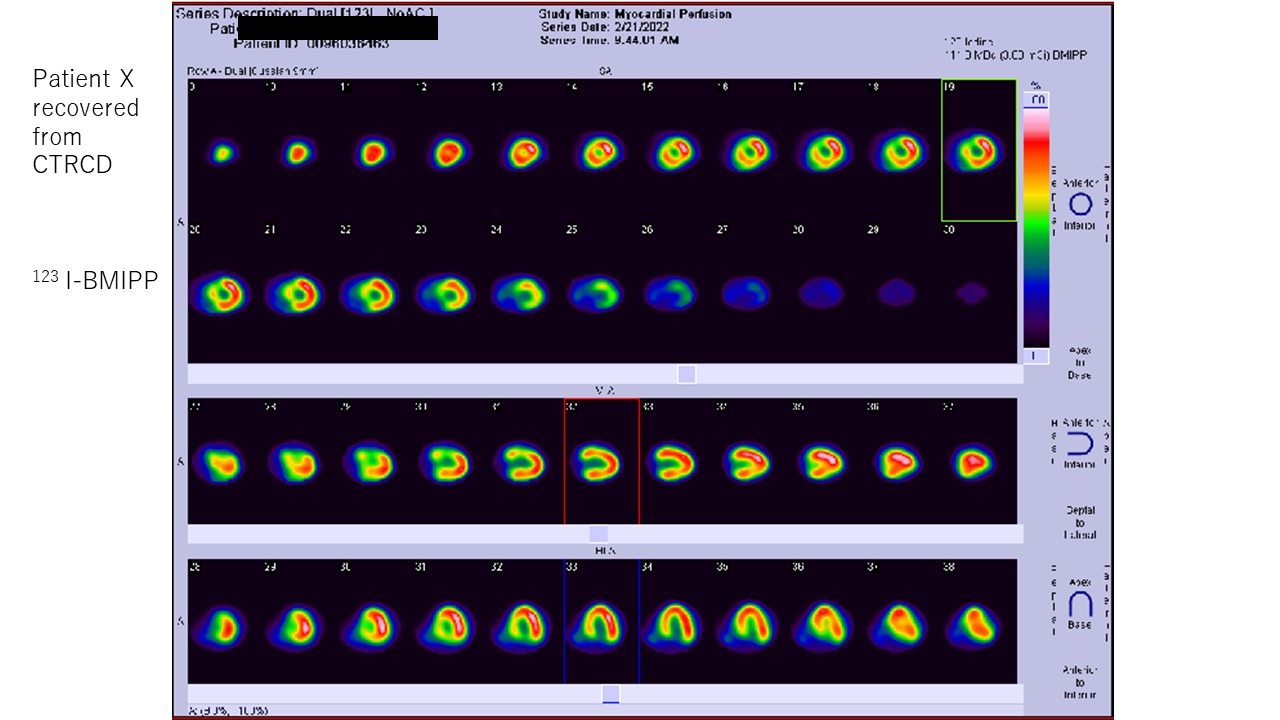

Supplement: Supplementary file 1 [file jimaging-10-00054-s001.zip › Supplement file 10.tif]

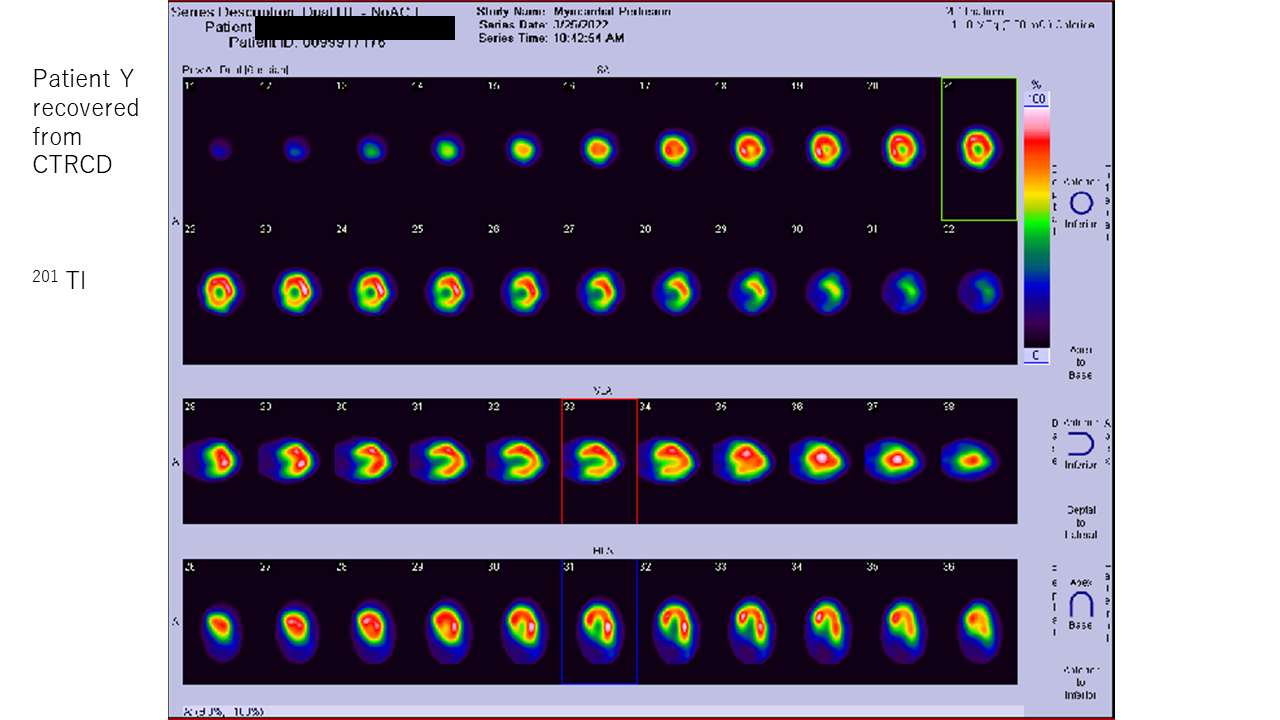

Supplement: Supplementary file 1 [file jimaging-10-00054-s001.zip › Supplement file 11.tif]

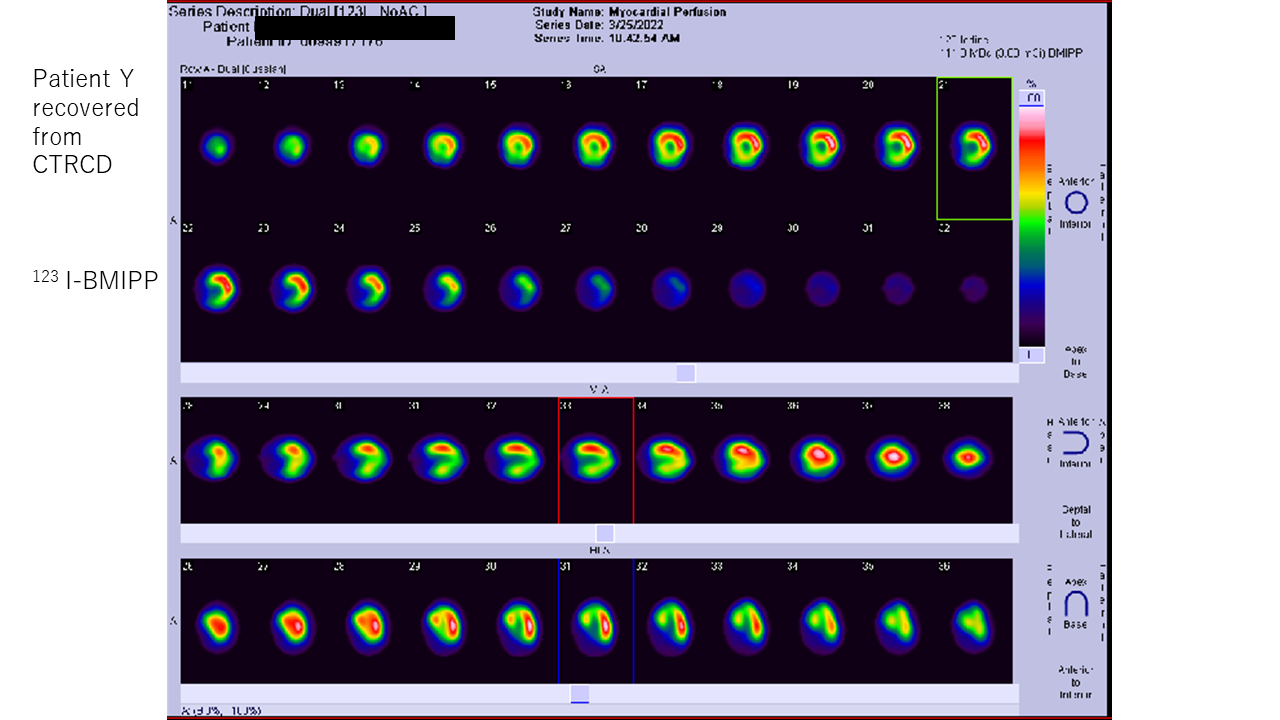

Supplement: Supplementary file 1 [file jimaging-10-00054-s001.zip › Supplement file 12.tif]

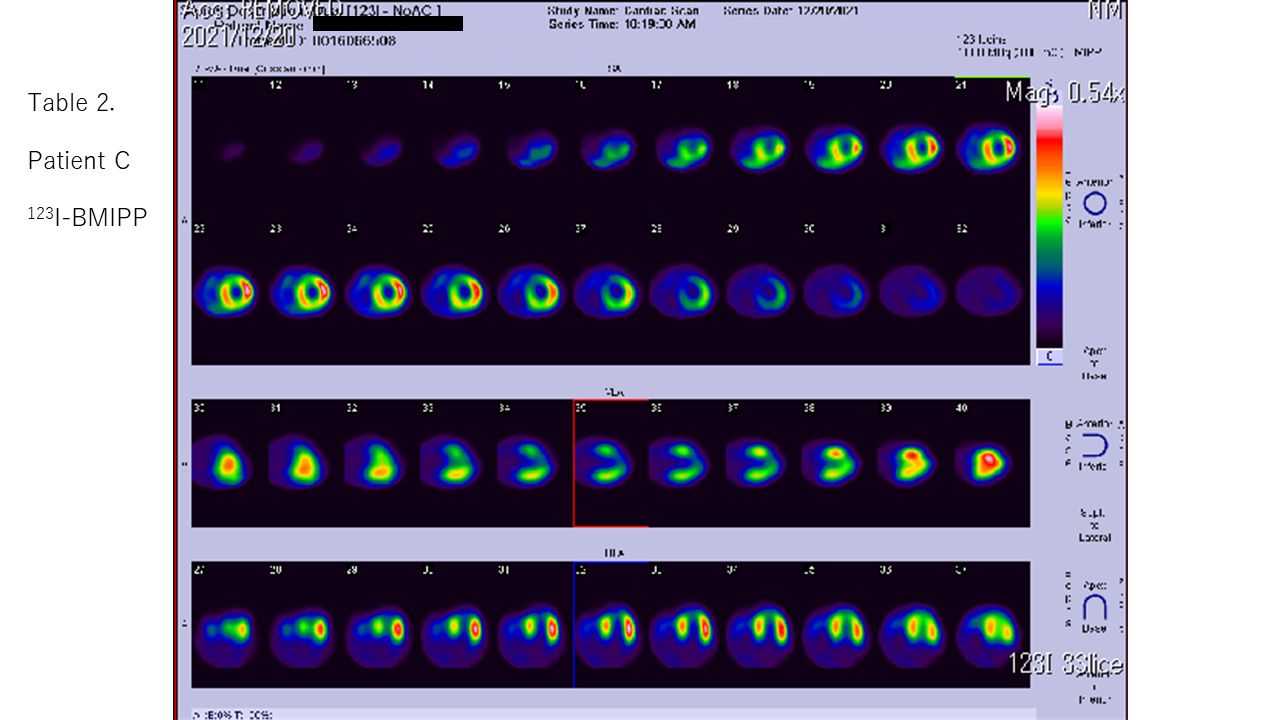

Supplement: Supplementary file 1 [file jimaging-10-00054-s001.zip › Supplement file 2.tif]

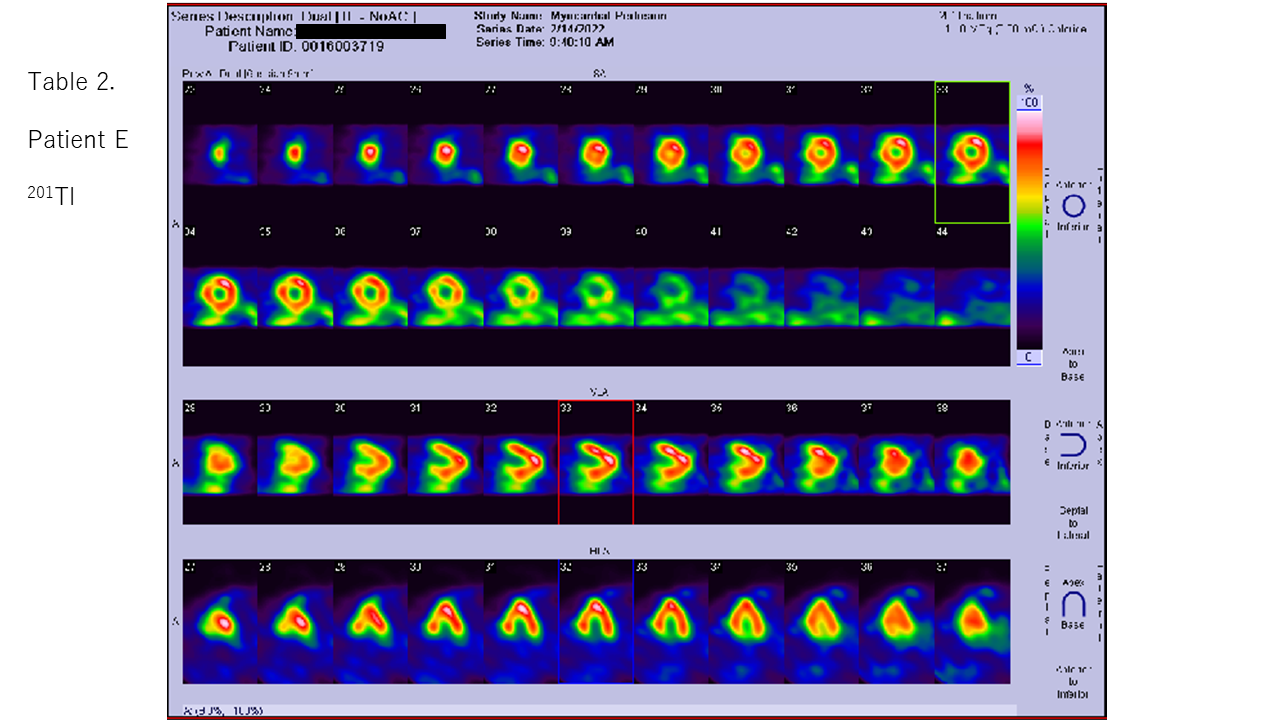

Supplement: Supplementary file 1 [file jimaging-10-00054-s001.zip › Supplement file 3.tif]

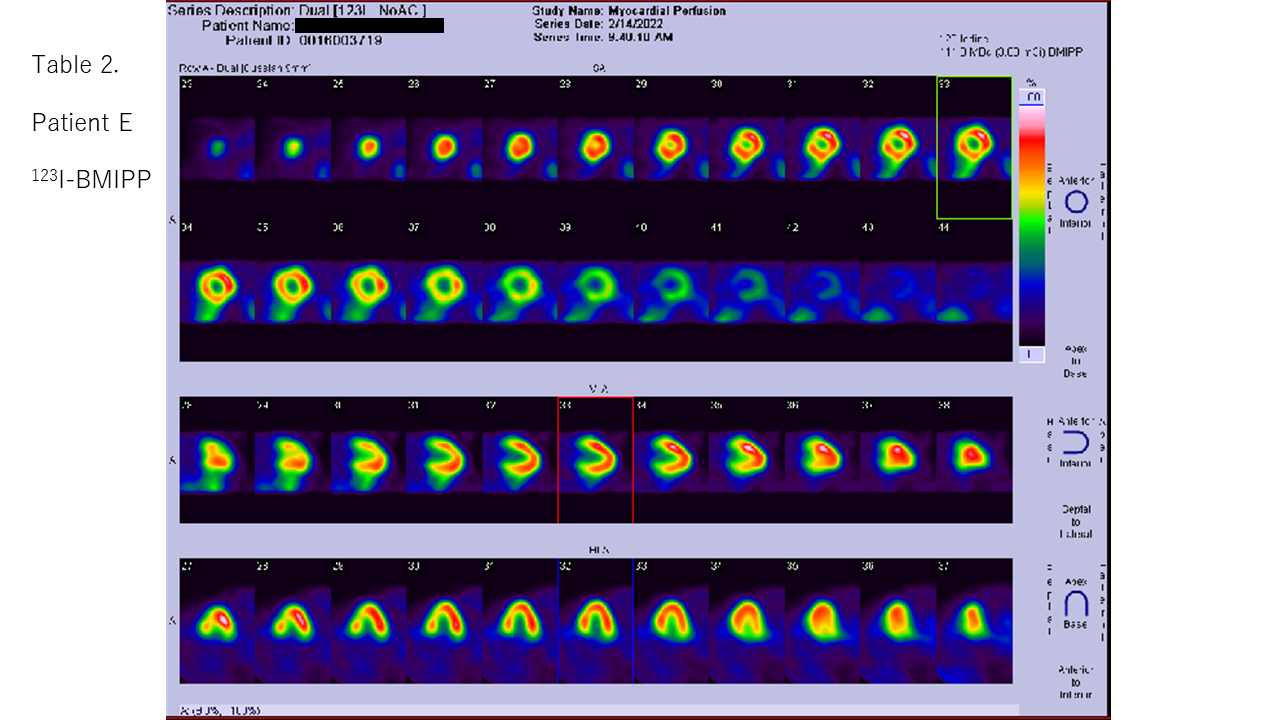

Supplement: Supplementary file 1 [file jimaging-10-00054-s001.zip › Supplement file 4.tif]

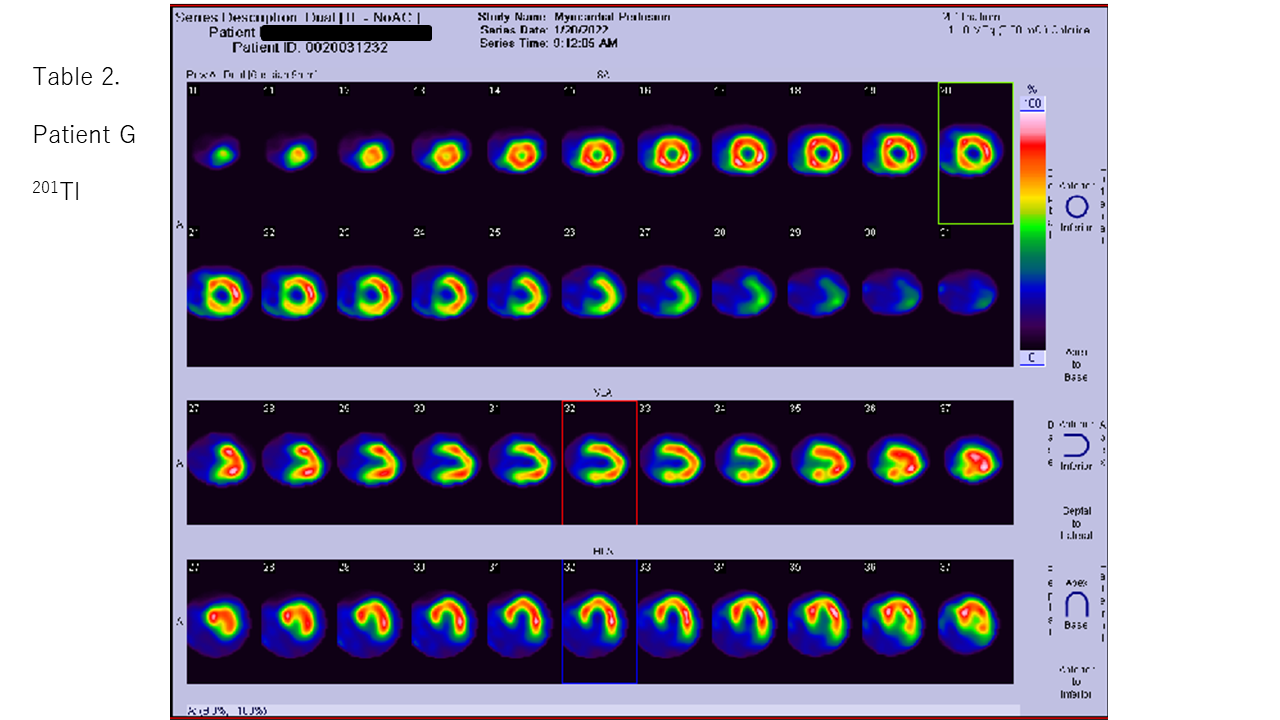

Supplement: Supplementary file 1 [file jimaging-10-00054-s001.zip › Supplement file 5.tif]

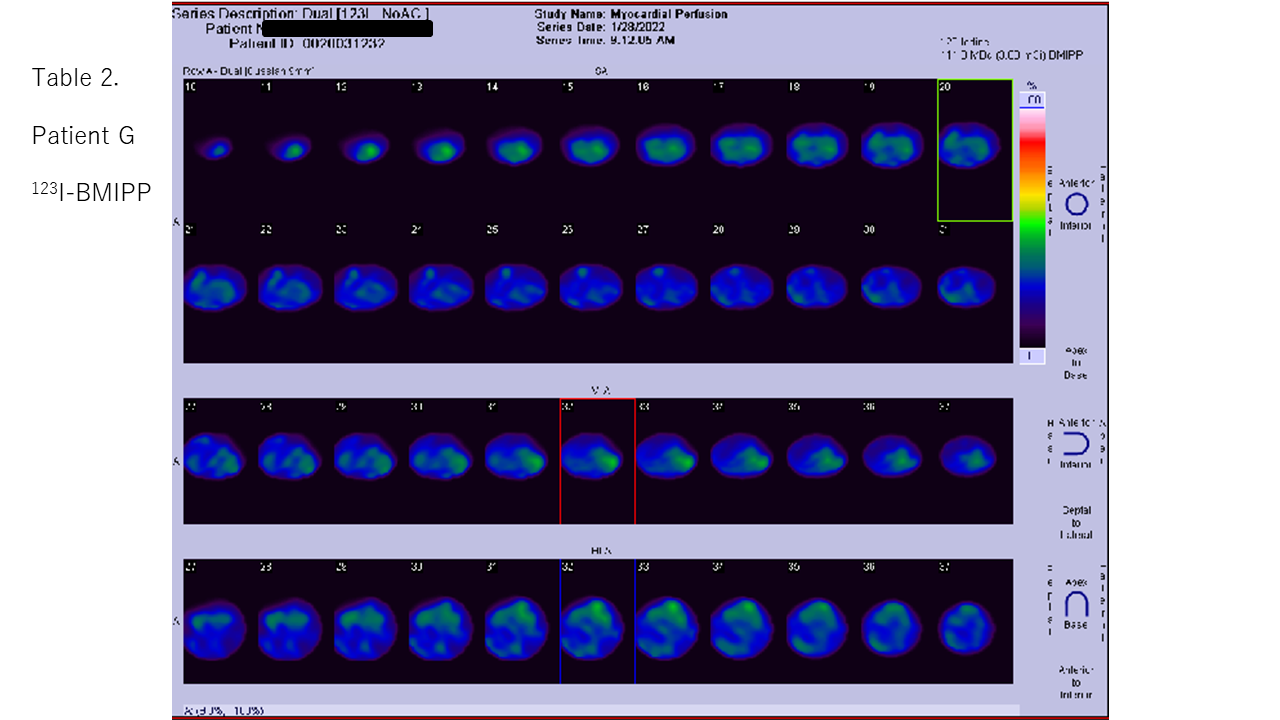

Supplement: Supplementary file 1 [file jimaging-10-00054-s001.zip › Supplement file 6.tif]

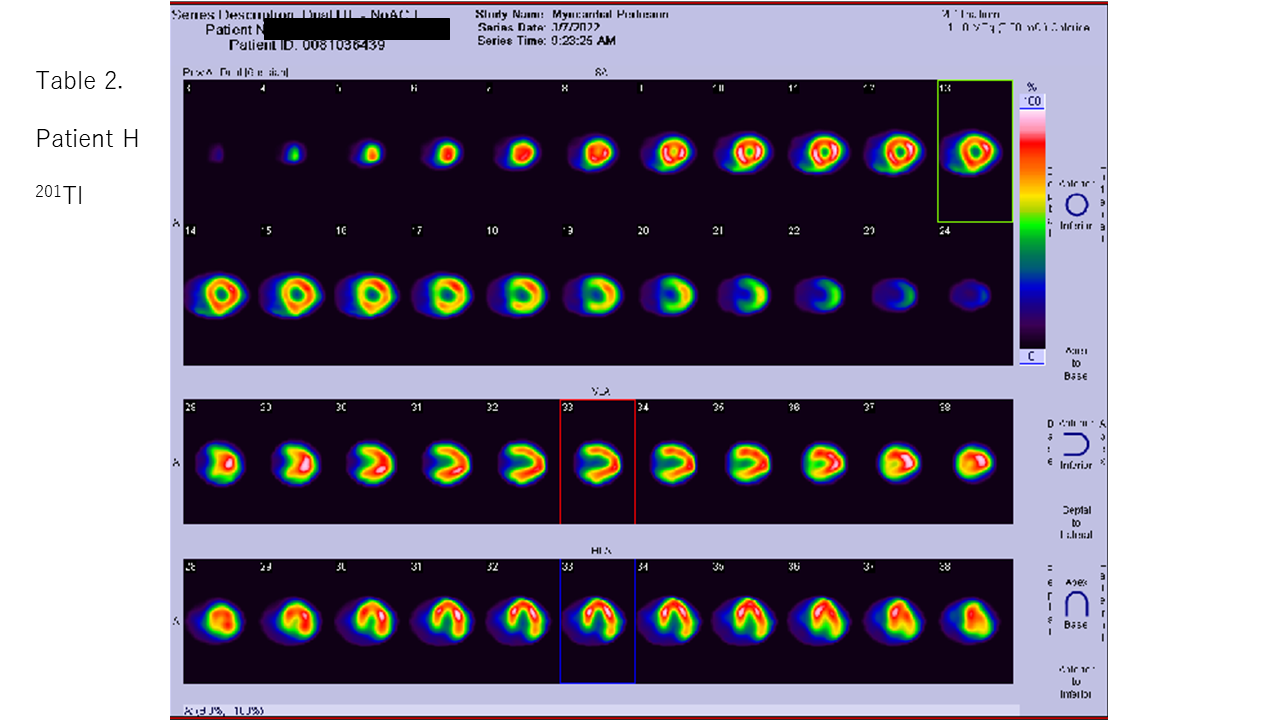

Supplement: Supplementary file 1 [file jimaging-10-00054-s001.zip › Supplement file 7.tif]

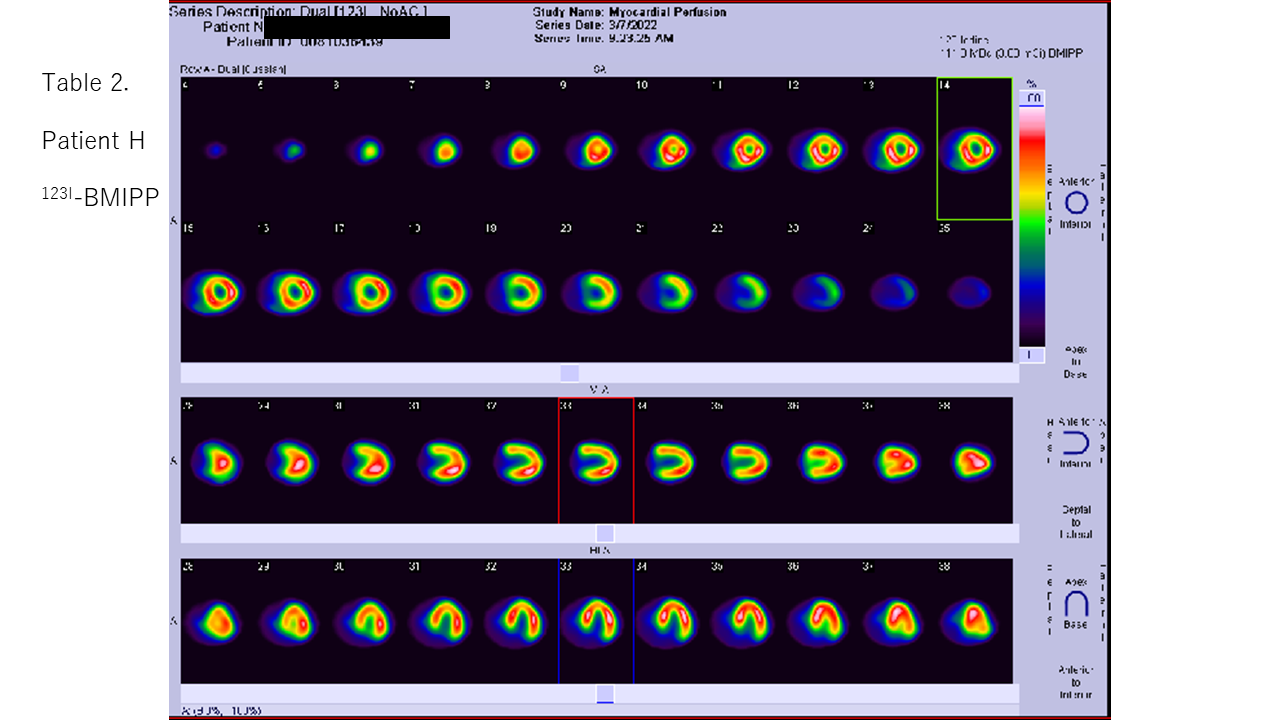

Supplement: Supplementary file 1 [file jimaging-10-00054-s001.zip › Supplement file 8.tif]

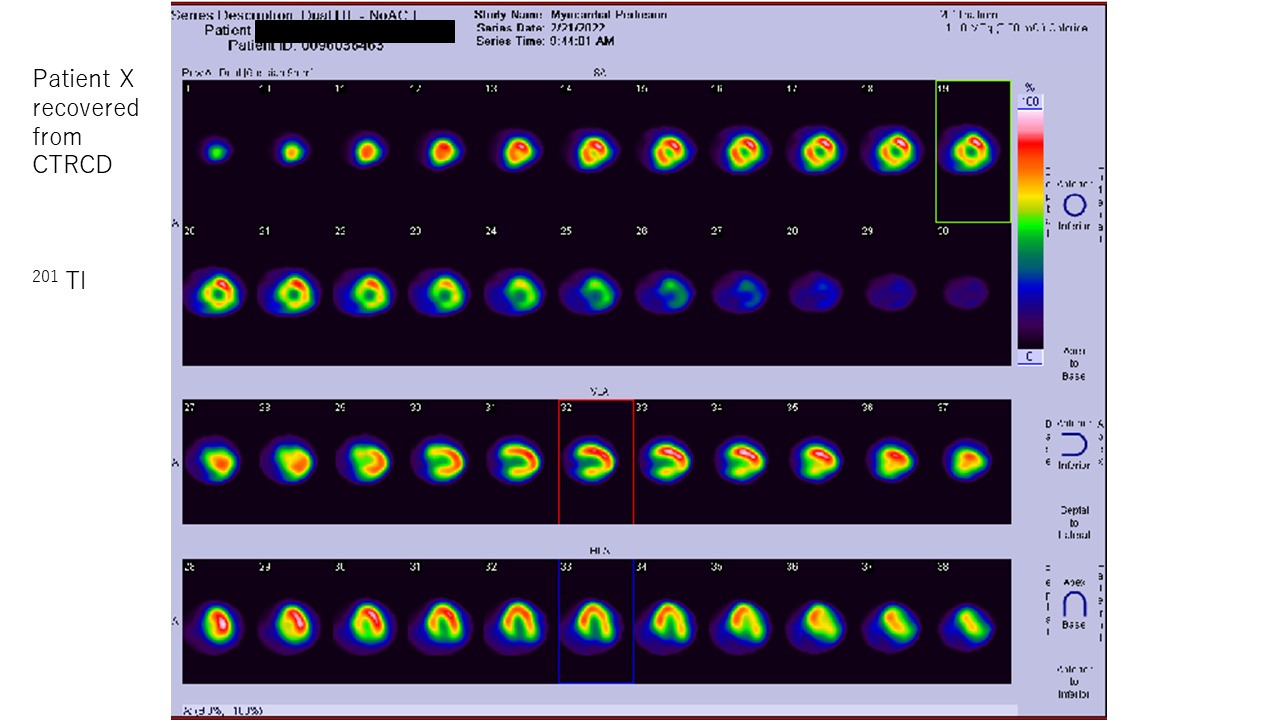

Supplement: Supplementary file 1 [file jimaging-10-00054-s001.zip › Supplement file 9.tif]
